# Supplementary material for: Neural computations underlying strategic social decision-making in groups
Source: Nat Commun. 2019 Nov 21;10:5287. doi: 10.1038/s41467-019-12937-5 (PMC6872737; doi:10.1038/s41467-019-12937-5)
Supplement: Supplementary file 1 — Supplementary Information [file 41467_2019_12937_MOESM1_ESM.pdf]

Supplementary Information

Neural computations underlying strategic decision-making during social interactions in groups

Park et al.

Supplementary Figures

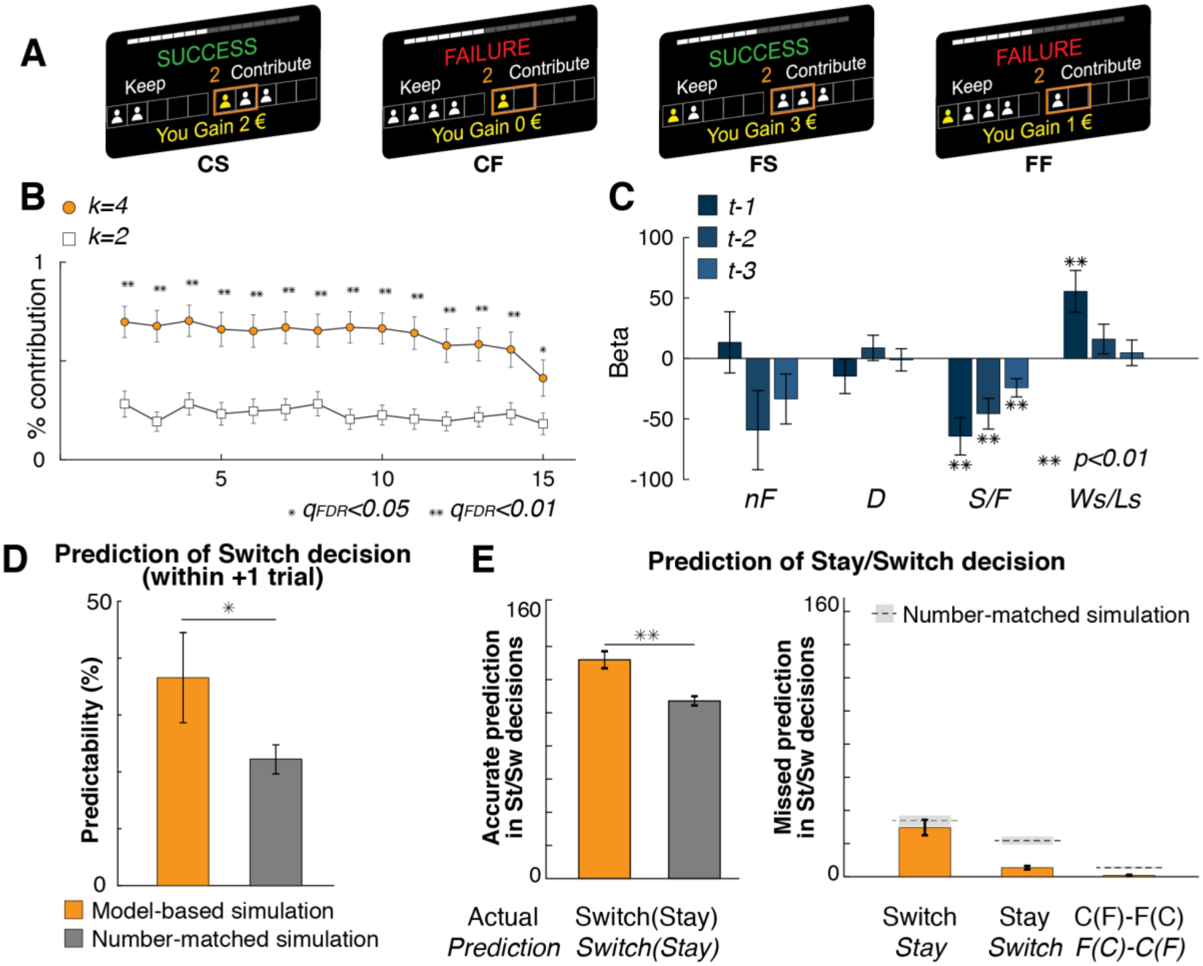

Supplementary Figure 1. A. The illustrated examples of each of four outcomes after the choice to Contribute (C) to the PGG or Free-riding (F), causing different payoffs (Success: S; Failure: F). (The choice of the participant is indicated by the yellow person and the choices of others are indicated by the white persons. The orange rectangle represents the decision threshold k needed to implement the PGG.) B. Average probability to contribute in each round ($2 \leq t \leq 15$) generated by the computational model. As in the actual decisions made by human participants, the weaker the volunteer's dilemma (k=4), the higher rate of contribution decisions was made by the social learning model during the PGG. Error bars indicate s.e.m; *: q<0.05 FDR corrected for multiple comparisons. C. Model-free analysis of the synthetic data. We regressed the behavioral decision on the number of free-riders (*nF*), previous decision (*D*), success or failure to generate the public goods (*S/F*), and win-stay and lose-switch strategy (*Ws/Ls*) in previous trials up to three trials back. Error bars indicate s.e.m; *: $p<0.05$, **: $p<0.01$ (mixed effect logistic regression). D. The percentage of accuracy of the model-based simulated decision in predicting the same switch decision that a participant made within the next one trial. The model-based simulated decision set used here is the same 12 folds cross-validation as shown in Fig S1 B and C. To examine how well switch decisions are predicted by the model-based simulation, we compared this with the baseline

prediction of switch decisions. To compute the baseline prediction, we measured how much the randomly shuffled data predict the switch/stay decisions while matching the number of contribution/free-riding decisions of each subject in each block (100 times iterations per block). The model-based simulation more accurately predicts the switch decision than the number-matched simulation (t_{24}=2.56, p=0.017, paired t-test). E. Average number of trials across participants in which the decision to switch from the previous strategy or to stay with the current strategy is accurately predicted by the synthetic decisions generated by the social learning model across 168 trials (except for the decision in the first trial of each block) (Left) and number of trials in which the model-based simulation differed from the actual switch/stay decision (right). While the upper labels indicate the actual behavior, the bottom italicized labels indicate its model predictions. That is, the three bars in the missed prediction graph (right) indicate the number of trials in the following order (from left to right): trials in which participants switch their strategy but the model predicts staying in the previous strategy; trials in which participants stayed in the previous strategy but the model predicts switching strategy; trials in which the model predicts switching strategy in the wrong direction. The social learning model not only generated series of switch/stay decisions that are more similar to those made by a participant than the number matched baseline (left; t_{24}=4.99, p<0.001), but had also lower miss predictions than the number matched baseline simulation (right). In the right panel, the first bar indicates the number of trials in which the model-based simulation made a stay decision when the participant made a switch decision; the second bar indicates the number of trials in which the model-based simulation made a switch decision when the participant made a stay decision; the third bar indicates the number of trials in which the model-based simulation had a switch decision in a different direction from the direction a participant made (e.g. the model switches from the contribution to free-riding, C-F while the participant switches from free-riding to contribution, F-C). The baseline prediction estimated from the number-matched simulation is shown in dotted line. Error bars indicate s.e.m.

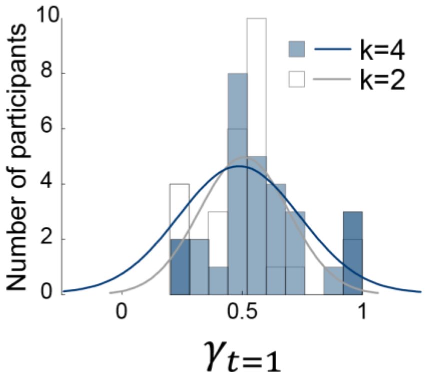

Supplementary Figure 2. Initial tendency to make a contribution (Contribution decisions in control blocks) For the first two games, participants made decisions in which they did not receive any feedback, whereby participants played either with a stronger (k=2), or with a weaker VD (k=4). Based on the assumption that participants initially expect that others contribute as much as they did, the contribution rates were inputted to set the initial belief of each participant about the decision of others at the first round. The distribution of the initial belief of each participant ($\gamma_{t=1}$) are shown here. To what extent an individual is willing to contribute to the group was measured by the rate of contribution decisions during the no-feedback. Based on the assumption that participants expect that others contribute with the similar probability, we infer the initial belief of participants about the probability that another will free-ride as the frequency of free-riding decisions of each of the participants while playing the PGG with no-feedback.

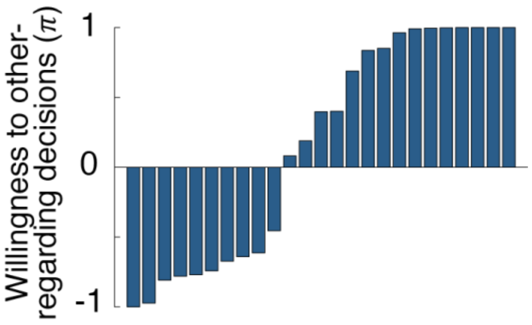

Supplementary Figure 3. The individual differences in willingness to make other-regarding decisions (π). The tendency of each participant to take the expected outcomes given to the others into their own utility has been captured as parameter, π. That is, with the larger π the participant has, the more likely to make an altruistic decision. Likewise, with the smaller π the participant has, the more likely to make a selfish decision.

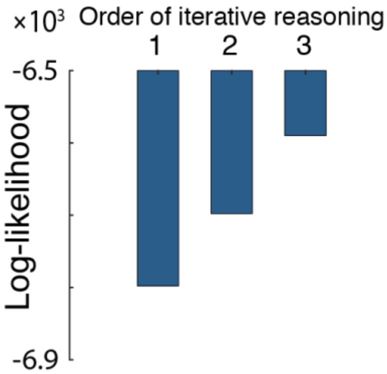

Supplementary Figure 4. The changes in quality of model fits (-2 log likelihood) resulting from changing the level of iterative reasoning in the social learning model. The 1st order beliefs model explains the decisions better than those of other higher order beliefs models.

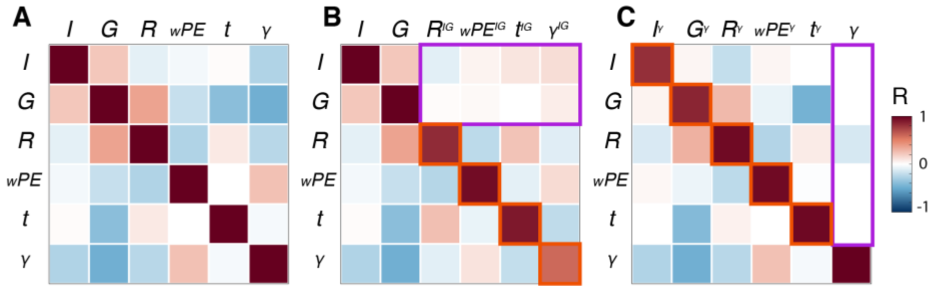

Supplementary Figure 5. We ran general linear model (GLM) analyses to identify the brain regions encoding the following computational variables: estimates of the individual utility (I), the group utility (G), and one's belief about the decision of others (γ). In addition, we allow these regressors to compete with other regressors: the reward allocated to the participant (R), the weighted prediction errors (wPE), and the trial number (t, to control the effects of the number of remaining trials). **A.** The mean cross-correlation among these regressors. **B.** For GLM1, to deal with multicollinearity of other regressors with the regressors of interests (I and G), we inputted the other regressors between regressors of non-interests (wPE, R, t, and γ) after regressing out their covariance with the regressors of interest by performing a partial correlation. The off-diagonal triangle shows the mean cross-correlation among the regressors inputted into the GLM1 – I, G, $\mathrm{wPE}^{\mathrm{IG}}$, R^{IG}, t^{IG} and γ^{IG}. Importantly, these regressors are still highly correlated with their original values (the diagonal highlighted in orange), while they do not correlate with I and G anymore (off-diagonal highlighted in purple). **C.** For GLM2, to deal with the multicollinearity of other regressors with the regressors of interests (γ), we inputted the other regressors between non-interests regressors (I, G, wPE, R, and t) after regressing out their covariance with the regressors of interest by performing a partial correlation. The off-diagonal triangle shows the mean cross-correlation among the regressors inputted into the GLM2 – I^{γ}, G^{γ}, wPE^{γ}, R^{γ}, t^{γ} and γ. Importantly, these regressors are still highly correlated with their original values (the diagonal highlighted in orange), while they do not correlate with γ anymore (off-diagonal highlighted in purple).

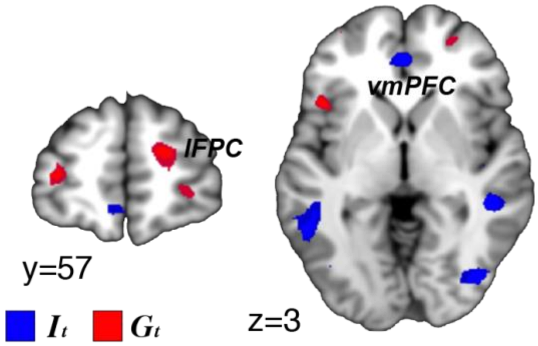

Supplementary Figure 6. Neural correlates of Individual utility (I_t) and Group utility (G_t) at the time of decision onset. Activity (in blue) in the ventromedial prefrontal cortex (vmPFC) at the time of decision on trial *t* inversely correlated with I_t. Activities (in red) in the right lateral frontopolar cortex (lFPC) and bilateral inferior parietal lobule (IPL) at the time of decision on trial *t* positively correlated with the estimated G_t. The statistical maps are thresholded at p<0.005, uncorrected (darker color). The lighter color map was thresholded at p<0.001, uncorrected.

Supplementary Tables

Brain area	MNI coordinate			Cluster size (k)	T-value
	x	y	z		
Brain areas encoding individual utility (I_t, Figure 3 associated)					
Ventromedial Prefrontal cortex (vmPFC)	0	56	-2	91	5.82
Insula (right)	36	-1	7	75	6.67
Brain areas encoding individual utility (G_t, Figure 3 associated)					
Lateral Frontopolar cortex (lFPC, right)	24	50	-8	167	6.17
Inferior Parietal lobule (iPL, Left)	-39	-58	37	137	6.06
Inferior Parietal lobule (iPL, Right)	45	-52	34	202	4.35
Precuneus	-6	-67	43	148	5.51
Brain areas tracking the free-riding decision of others (γ_t; Figure 4 C associated)					
Anterior cingulate cortex gyrus (ACCg)	0	20	31	126	4.61
Brain areas tracking the free-riding decision of others ($1-\gamma_t$; Figure 4 D associated)					
Temporoparietal junction (TPJ, left)	-48	-55	37	62	5.42
Temporoparietal junction (TPJ, right)	51	-55	40	60	5.81
Brain areas engaged in the arbitration between different strategies (Figure 5 A associated)					
Anterior Cingulate cortex (ACC)	0	17	31	67	5.23
ventrolateral Prefrontal Cortex (vlPFC, right)	39	26	13	69	4.35

Supplementary Table 1. Brain areas involved in the strategic decision-making during social interactions. MNI coordinates show peak activations for each cluster. We report results corrected with FWE for multiple comparisons in the cluster level ($p < 0.05$) on the basis of an initial uncorrected threshold at $p < 0.001$.

Brain area	MNI coordinate			Cluster size (k)	T-value
	x	y	z		
Positive connectivity (Figure 5 B associated)					
lFPC (right)	30	50	1	51	4.58
Negative connectivity (Figure 5 B associated)					
vmPFC	9	50	-8	27	4.66

Supplementary Table 2. Brain areas showing stronger functional connectivity with areas arbitrating between two strategies and modulated by the probability of contribution. MNI coordinates show peak activations for each cluster. We report results corrected within the anatomically defined regions of interests (ROIs) for multiple comparisons in the small-volume level ($p < 0.05$) on the basis of an initial uncorrected threshold at $p < 0.001$.

Supplementary Discussion

Optimal decisions in the public goods game confronted with the volunteer's dilemma

When one or few volunteers can produce the public goods, participants face the volunteer's dilemma. That is, in addition to the risk that the public good is not produced, individuals in this game also face a risk that their contribution is wasted when the contribution from other members are enough to produce the public good [1]. In the classical PGG, free-riding is the dominant strategy which is also the subgame perfect equilibrium in finite interactions for individuals [2–4], while making a contribution to the group increases the benefits for the group from the public goods. When facing the volunteer's dilemma, the outcome of one's decision is determined by the decision of others. In this game, neither the pure strategy consisting of voluntary contribution or free-riding is dominant over the other. Instead, the mixed-strategy equilibrium is found in which each pure strategy is chosen with a certain probability (a mixed strategy is an assignment of a probability to each pure strategy).

The mixed-strategy equilibrium is defined where the expected utility of contributing equals to that of free-riding. Moreover, the equilibrium decision of the probability to choose free-riding, γ, should be same for every participant. Taking these aspects into account, a previous study [5] has shown that the mixed-strategy equilibrium in the PGG in which at least k individuals volunteer is required to produce the public goods in a group of N people ($N > k$) (Supplementary Equation 1). Specifically, when everybody chooses the free-riding decision with the mixed-strategy equilibrium probability, γ, the probability, $\Gamma_{N\text{-}k}$ that $N\text{-}k$ other individuals free-ride among $N\text{-}1$ (all other players except the player himself) depends on the ratio of the individual contribution costs ($c > 0$) over the group benefits ($R > c$).

$$\frac{c}{R} = \Gamma^{N-k} = \binom{N-1}{N-k} \gamma^{N-k} (1-\gamma)^{k-1}$$

Equation. 1

This study shows that the probability that $N\text{-}k$ people free-ride increases with the cost of individual contribution (c) and decreases with the benefits (R) if the group gets the benefits from the public goods. Moreover, γ, the probability to free-ride, increases with N and decreases with k, indicating that the public goods can be generated with a small number of volunteers in a larger group, each member relying more on others' contribution.

Updating the social prediction errors

When the players update their belief about the intention of others for future decisions, we hypothesized that the update process is guided not only by the social prediction error (PE_S)but also the reward prediction error (PE_R) based on the following rationale. In groups, participants' belief about the decision of others might be biased by whether their group was successfully generating the public goods or not. That is, participants may believe that others are more likely to be cooperative when getting the benefits and less likely to contribute when they cannot expect

the group reward. Moreover, the closer the prediction about the number of free-riders to *N-k*, the higher the uncertainty the participants have in the probability to win the public goods (when $\Gamma_{N\text{-}k}$ is high, $p(R|\,\gamma) \approx .5$). Importantly, when their expectation about the number of the free-rider is close to *N-k*, participants have a larger reward prediction error, despite the same size of PE_S. As the left panel in **Figure 2C** shown, with increase in the probability that an individual member free-rides (γ), the probability that the group produces the public goods decreases ($p(R = 2MU|\,\gamma) = \sum_{i=0}^{N-k} \Gamma^i$). Specifically, the probability that the group gets the reward follows the cumulative distribution function (CDF) of the binomial distribution in which individual members free-ride with probability *γ*, which suggests that the reward probability does not linearly decrease with *γ*. Instead, when having the same level of PE_S, participants experience a larger PE_R when their expectation about the number of other free-riders was close to *N-k* (where $\gamma \approx \underset{\gamma}{argmax}\, \Gamma^{N-k}$).

Taking this into account, we tested the hypothesis that the learning rate of the decision of others tends to increase as a function of the absolute magnitude of the recent monetary prediction error. The results showed that participants increase their learning rate of the social prediction error in proportion to the magnitude of the monetary prediction error, adapting their beliefs to the recent observations when experiencing a violation of one's expectation of public goods.

Decay in contributions in a PGG with the same partners

Previous behavioral economics studies using public goods games have found a consistent pattern of contribution decisions. That is, the mean contribution rate is high on the first round but declines over time [6,7]. Moreover, once the PGG restarts, the contributions jump up for new partners. Consistent with these previous findings, we found that the mean contribution rate gradually decayed with the progress of the PGG under volunteer's dilemma. We further compared the influences of volunteer's dilemma on the degree of decays. To do this, we estimated the slopes of the decays in the mean contribution rate of each participant using a linear regression analysis. Then, we compared the two slopes between games under the influences of the two levels of volunteer's dilemma. We found that the contribution rates decayed more steeply while confronted with a stronger volunteer's dilemma than compared to those when confronted with a weaker volunteer's dilemma (t_{24}=2.16, p=0.04, paired t-test).

Neural mechanisms of strategy selection in social interactions

The functional connectivity between the brain areas selectively engaged for the event predicting the strategy switch (vlPFC and ACC) decreased with the vmPFC and increased with the lFPC when the probability of contribution is increasing ($\Delta Q > 0$) at the time of feedback. This finding suggests that the brain computes the expected utility by integrating the individual utility encoded in the vmPFC and the group utility encoded in the lFPC. When expected utility of the current strategy is lower than that of alternative strategy, these brain regions exhibited changes in functional interactions to the vlPFC and ACC to guide a switch between strategies.

Our findings provide a framework that is distinct from −but compatible with− previous works examining conditional cooperators [8,9]. In these studies, participants continued to contribute until it was mutually beneficial. Extending these findings, our study provides evidence that the human brain constructs a predictive model about the decision of others and computes the expected returns of future interactions to evaluate the current strategy. Such neural computations allow for adaptive and efficient decision-making during social interactions in which one's optimal strategy varies with the intention of others, a hallmark of human social cognition.

Supplementary Methods

Public goods game confronted with different levels of the volunteer's dilemma

In the Public goods game (PGG) confronted by the volunteer's dilemma, bystanders decide independently on whether to sacrifice themselves for the benefit of the group. On one hand, because of the cost of volunteering, individuals have a greater incentive for freeriding than to sacrifice oneself for the group. On the other hand, nobody will benefit from the group rewards if no one volunteers.

We modulated the level of the volunteer's dilemma by changing the threshold k of this minimum number of contributors required to generate the public goods. That is, we induced a stronger volunteer's dilemma in trials requiring only a few volunteers to generate the public goods (e.g. a group faces a stronger volunteer's dilemma when k=2 compared to when k=4), thereby increasing the probability that one's contribution is not serving to generate the public goods but is instead wasted. Specifically, during half of the games, public goods were produced when there were at least two volunteers who contributed (k=2), and during the other half of the games, public goods were produced when there were at least four volunteers who contributed ($k=4$). **Supplementary Figure 1A** shows all the potential outcomes when a participant contributes to the group (the yellow icon is the participant). When the group gets the rewards, one's contribution serves to generate the public goods only when N-k people free-ride. In the other cases, one's contribution does not help to produce the public goods. When fewer volunteers are required to produce the public goods, there is a higher risk that one's contribution is wasted, thereby inducing a strong volunteer's dilemma.

The decision of others

The decision of others was determined by the following function given the size of the group, N, the number of remaining interactions, T-$t+1$, the minimum ratio of contributors to generate public goods, K ($K=k/N$), the previous decision of participants (C_{t-1}^{i}; it was 1 if they made a contribution), and the proportion of contributors among others in the previous round ($\bar{C}_{t-1}^{N-1}$).

$$logit(\bar{C}_t^{N-1}) = \beta C_{t-1}^{i} + (1-\beta)\left\{\left(\frac{1-K^{T-t+1}}{1-K}\right)\bar{C}_{t-1}^{N-1} - K\right\}$$

Equation. 2

Note that this function has a parameter β which reflects to what extent others copied the decision of participants. By fitting the model to the behavioral data of actual human interactions [10], β was determined within the range $0.15 \leq \beta \leq 0.35$. Moreover, the effects of successful cooperation decay with the number of remaining interactions (T-$t+1$). In the first round (t=1), others' decisions were determined by the contribution rate of the participant, which was measured while she made no-

feedback PGGs (because the computer did not have a previous decision history of the participant).

The computer agent was programmed to interact with the participants' decisions themselves in an ecological manner. This has been used in other studies of social interactions, and it allowed us to ensure that every participant played against agents whose decision was based on the same algorithm [8]. In doing so, participants were more likely to interact with cooperative fellow members when they contributed their resources in the previous round and after the group successfully generated a public good.

Model validation and comparison

Given that the social learning model outperforms the alternative models, we tested further whether the social learning model accurately predicts the series of decisions made during a PGG (test-set) from the independent data (training-set). To do this, we conducted a leave-one-block-out cross-validation approach such that the decision made for the N-th PGG is predicted based on the parameters estimated by fitting the model to decisions made for the other 11 PGGs except for the N-th PGG of each participant. This process was repeated 12 times. In doing so, we simulated new data using our parameters fitted to an independent dataset. First, the social learning model predicts that for the PGG with stronger volunteer's dilemma, participants are less likely to contribute to the PGG compared to the weaker volunteer's dilemma across trials. The mean contribution rates in the model-predicted dataset are shown in **Supplementary Figure 1B**. Second, we also performed a model-free analysis of the decisions generated by computational models. We found that the model free characteristics that we observed in the actual behavior were largely recapitulated in the model-generated behaviors (**Supplementary Figure 1C**). When a participant switches their previous decision, the model also predict the same change in 68.4±0.10% of chance within next 3 trials, which is even founded in the model-based simulation with cross-validation in 42.4±0.09% chance. We also found that, in the model-based simulation, the decisions of each of participants about whether they switch their decision to the contribution or free-riding or whether stay to the previous strategy were accurately predicted for 131.9±5.1 (s.e.m.) trials among 168 in total (except for the first decision in each block of PGG). To test how well these decisions in the model-based simulation reflect the actual sequence of switch/stay decisions made by a participant, we compared the model-based simulation to the other control simulation. The control simulation was made 100 times iteration while matching the number of contribution/ free-riding decisions that the model-based simulation made during a block of PGG. We found that the agent in the model-based simulation is more likely to make the same switch/stay decisions at the same trial that the actual decision was made by a participant, which was significantly above the baseline, the other number matched control simulation (t_{24}=4.99, $p<0.001$, paired t-test; **Supplementary Figure 1D**).

Last, we tested the contribution of free-parameters of social learning models to the quality of the fit. The social learning model contained four free parameters, α, θ, π, and λ, respectively associated with the learning rate on the estimated decision of others, the weight on the reward prediction error, one's altruistic tendency, and the subjective contribution cost. We tested whether

these parameters were necessary to explain strategic decision-making by investigating whether removing any of these parameters causes a decrease in the quality of the fit. This was assessed with the changes in log likelihood using the same cross-validation procedure (leave-one-block-out). We found that the goodness of fit decreases when fixing any of four parameters as a constant value (**Figure 1F**).

Social contextualization

To increase the credibility of the cover story, participants heard voices during the inter-game interval, while seeing the screen with the message, ‘waiting for starting a new game with new partners'. Participants heard the voice instruction that was recorded from the behavioral experiment room. Moreover, after making decisions for some trials, participants were asked to make their decision again after seeing an error message, ‘some participants did not make a decision’. After the experiment finished, participants answered a questionnaire (see below). We analyzed data of the participants who believed that they were interacting with real participants simultaneously. The mean social contextualization score (normalized) ± standard deviation (S.D.) was .82 ± .16 (n=30). The participants whose score was lower than 3×s.d. from the mean were not included for the analyses (4 participants, normalized mean score=.24±.02).

Instructions: please read the following questions and tick on the scale displayed how much you strongly agree (10) or disagree (1).

1. Sometimes, other players changed their strategy based on my previous decisions.
2. During the experiment, I interacted with other players without significant time delays.
3. Some players were more cooperative than other players.
4. Sometimes, other players changed their strategy in order to increase the monetary reward for the group.
5. Some of other players care less about the group earning, instead they care about their monetary outcome.
6. Sometimes, I changed my strategy based on previous decisions of other players.
7. Sometimes, I changed my strategy in order to increase the monetary reward for the group.
8. When the feedback is given at the end of every trial, I predict the next decision of other players.
9. When the feedback is given, another player might predict the next decision of the others.
10. While playing this game, other human players simultaneously made decisions with me.

Supplementary References

1. Diekmann, A. Volunteer's dilemma. *J. Conflict Resolut.* **29**, 605–610 (1985).
2. Andreoni, J. Why free ride? Strategies and learning in public goods experiments. *J. Public Econ.* **37**, 291–304 (1988).
3. Palfrey, T. R. & Rosenthal, H. Participation and the provision of discrete public goods: a strategic analysis. *J. Public Econ.* **24**, 171–193 (1984).
4. Benoit, J.-P. & Krishna, V. Nash equilibria of finitely repeated games. *Int. J. Game Theory* **16**, 197–204 (1987).
5. Archetti, M. Cooperation as a volunteer's dilemma and the strategy of conflict in public goods games. *J. Evol. Biol.* **22**, 2192–200 (2009).
6. Fischbacher, U. & Gächter, S. Social Preferences, Beliefs, and the Dynamics of Free Riding in Public Goods Experiments. *Am. Econ. Rev.* **100**, 541–556 (2010).
7. Cadsby, C. B. & Maynes, E. Gender and free riding in a threshold public goods game: Experimental evidence. *J. Econ. Behav. Organ.* **34**, 603–620 (1998).
8. Suzuki, S., Niki, K., Fujisaki, S. & Akiyama, E. Neural basis of conditional cooperation. *Soc. Cogn. Affect. Neurosci.* **6**, 338–347 (2011).
9. Fischbacher, U., Gächter, S. & Fehr, E. Are people conditionally cooperative? Evidence from a public goods experiment. *Econ. Lett.* **71**, 397–404 (2001).
10. Park, S. A., Jeong, S. & Jeong, J. TV programs that denounce unfair advantage impact women's sensitivity to defection in the public goods game. *Soc. Neurosci.* **8**, 568–582 (2013).
