# Supplementary material for: Neural computations underlying strategic social decision-making in groups
Source: Nat Commun. 2019 Nov 21;10:5287. doi: 10.1038/s41467-019-12937-5 (PMC6872737; doi:10.1038/s41467-019-12937-5)
Supplement: Supplementary file 3 — Reporting Summary [file 41467_2019_12937_MOESM3_ESM.pdf]

nature research

Corresponding author(s): NCOMMS-18-30157

Reporting Summary

Nature Research wishes to improve the reproducibility of the work that we publish. This form provides structure for consistency and transparency in reporting. For further information on Nature Research policies, see Authors & Referees and the Editorial Policy Checklist.

Statistical parameters

When statistical analyses are reported, confirm that the following items are present in the relevant location (e.g. figure legend, table legend, main text, or Methods section).

n/a	Confirmed	
☐	☒	The exact sample size (n) for each experimental group/condition, given as a discrete number and unit of measurement
☐	☒	An indication of whether measurements were taken from distinct samples or whether the same sample was measured repeatedly
☐	☒	The statistical test(s) used AND whether they are one- or two-sided<br>*Only common tests should be described solely by name; describe more complex techniques in the Methods section.*
☐	☒	A description of all covariates tested
☐	☒	A description of any assumptions or corrections, such as tests of normality and adjustment for multiple comparisons
☐	☒	A full description of the statistics including central tendency (e.g. means) or other basic estimates (e.g. regression coefficient) AND variation (e.g. standard deviation) or associated estimates of uncertainty (e.g. confidence intervals)
☐	☒	For null hypothesis testing, the test statistic (e.g. F, t, r) with confidence intervals, effect sizes, degrees of freedom and P value noted<br>*Give P values as exact values whenever suitable.*
☒	☐	For Bayesian analysis, information on the choice of priors and Markov chain Monte Carlo settings
☒	☐	For hierarchical and complex designs, identification of the appropriate level for tests and full reporting of outcomes
☐	☒	Estimates of effect sizes (e.g. Cohen's d, Pearson's r), indicating how they were calculated
☐	☒	Clearly defined error bars<br>*State explicitly what error bars represent (e.g. SD, SE, CI)*

Our web collection on statistics for biologists may be useful.

Software and code

Policy information about availability of computer code

Data collection	Brain Imaging was performed on a 3T Philips Achieva MRI scanner. The stimuli presentation was made using Presentation software (14.0).
Data analysis	Behavioral data was analyzed using Matlab 2016a. The computational modeling was performed using Matlab 2016a. Brain imaging data were analyzed using SPM8 in combination with Matlab 2016a.

For manuscripts utilizing custom algorithms or software that are central to the research but not yet described in published literature, software must be made available to editors/reviewers upon request. We strongly encourage code deposition in a community repository (e.g. GitHub). See the Nature Research guidelines for submitting code & software for further information.

Data

Policy information about availability of data

All manuscripts must include a data availability statement. This statement should provide the following information, where applicable:

- Accession codes, unique identifiers, or web links for publicly available datasets
- A list of figures that have associated raw data
- A description of any restrictions on data availability

Anonymized behavioral data and the codes that support the findings are available in the repository in center for open science, https://osf.io/rdvsz/?

view_only=6326ed83ec3d46c196a6f0f9bd4feb85. Unthresholded group-level statistical maps are available on NeuroVault (https://neurovault.org/collections/QXJNIAXH/).

Field-specific reporting

Please select the best fit for your research. If you are not sure, read the appropriate sections before making your selection.

☒ Life sciences ☐ Behavioural & social sciences ☐ Ecological, evolutionary & environmental sciences

For a reference copy of the document with all sections, see nature.com/authors/policies/ReportingSummary-flat.pdf

Life sciences study design

All studies must disclose on these points even when the disclosure is negative.

Sample size	We used a sample size greater than the norm for the field (n=25 > 20). Moreover, our results are not depends on the individual differences across participants.
Data exclusions	We originally recruited 30 participants. Because of excessive head movement and responses in the post-scanning questionnaire, data of five participant was not included for analysis.
Replication	We replicated the behavioral parameters using cross-validation. We used independent samples to estimate the parameters and predict the behavior with the test set.
Randomization	Subjects were not allocated to different treatment groups. All experimental conditions were presented randomly interleaved.
Blinding	The within-subject design that we used in this study did not require blinding.

Reporting for specific materials, systems and methods

Materials & experimental systems

n/a	Involved in the study	
☒	☐	Unique biological materials
☒	☐	Antibodies
☒	☐	Eukaryotic cell lines
☒	☐	Palaeontology
☒	☐	Animals and other organisms
☐	☒	Human research participants

Methods

n/a	Involved in the study	
☒	☐	ChIP-seq
☒	☐	Flow cytometry
☐	☒	MRI-based neuroimaging

Human research participants

Policy information about studies involving human research participants

Population characteristics	25 individuals (mean age 22.48 years old±0.33 (SD), 13 women) completed the study. Based on self-reported questionnaires, none of participants reported a history of neurological or psychiatric disorders. All subjects had normal or corrected to normal vision.
Recruitment	Students of University of Parma, Italy were recruited based on advertisement flyers.

Magnetic resonance imaging

Experimental design

Design type	Event-related, randomized trial sequence
Design specifications	Each subjects completed 12 runs with 15 trials each between two randomly interleaved choice conditions according to the decision threshold, k. The inter trial interval was jittered in 2 to 5 seconds.
Behavioral performance measures	In each trial, 1) a binary decision whether to make contribution or free-riding to the group, 2) reaction time, 3) satisfaction rating were measured.

Acquisition

Imaging type(s)	T1-weighted MP-rage, T2*-weighted gradient-echo planar imaging
Field strength	3 Tesla
Sequence & imaging parameters	The imaging parameters were as follows: repetition time (TR), 2500 ms; echo time (TE), 30 ms; acceleration factor 2, bandwidth 3906 Hz/PIXEL matrix 96x96, field of view (FOV), 205 × 205 mm2; 41 contiguous slices were acquired in interleaved order, slice thickness, 2.8 mm + 0.7 mm gap. The imaging parameters for the 3D IR-prepared FSPGR T1 weighted anatomical scan were as follows: TR, 8500 ms; TE, 3.2 ms; FOV, 256 × 256 mm2; matrix 256x256; slice thickness, 1 mm; total slices, 156, bandwidth 244 Hz/PIXEL.
Area of acquisition	Whole-brain

Diffusion MRI ☐ Used ☒ Not used

Preprocessing

Preprocessing software	SPM8
Normalization	Each structural image was segmented into gray matter, white matter and cerebral spinal fluid images using a nonlinear deformation field and mapped on a template. The deformations were further applied to both structural and functional images to create new images spatially normalized to Montreal Neurological Institute (MNI) space.
Normalization template	SPM8 MNI template
Noise and artifact removal	6 motion correction parameters were estimated from the realignment procedure and were entered as nuisance covariates. The onset time of any button press was entered as stick function to remove the potential motion effects.
Volume censoring	A participant who had more than 3mm movement from an EPI to the next image was not included for the analysis.

Statistical modeling & inference

Model type and settings	Mass univariate; fixed-effects within subject to combine fMRI data across runs; random-effects across subjects for second-level analyses.
Effect(s) tested	The group level effects was tested with one-sample t-test using SPM8. The statistical inference was conducted using Gaussian random field theory as implemented in SPM8 to obtain clusters satisfying P<0.05, family-wise error (FWE) corrected at a cluster-defining threshold of P<0.001 uncorrected.

Specify type of analysis: ☒ Whole brain ☐ ROI-based ☐ Both

Statistic type for inference (See Eklund et al. 2016)	Voxel-wise inference
Correction	The clusters satisfying P<0.05, family-wise error (FWE) corrected at a cluster-defining threshold of P<0.001 uncorrected.

Models & analysis

n/a	Involved in the study
☐	☒ Functional and/or effective connectivity
☒	☐ Graph analysis
☒	☐ Multivariate modeling or predictive analysis

Functional and/or effective connectivity	The Psycho-Physiological Interaction (PPI) was conducted using SPM8
